# Analysis of Infection Transmission Routes through Exhaled Breath and Cough Particle Dispersion in a General Hospital

**DOI:** 10.3390/ijerph19052512

**Published:** 2022-02-22

**Authors:** Minji Jung, Woong June Chung, Minki Sung, Seongmin Jo, Jinkwan Hong

**Affiliations:** 1 Department of HVAC System and Fire Protection Engineering, Gachon University, Seongnam 13120, Korea; mjjung06@gmail.com (M.J.); wjchung@gachon.ac.kr (W.J.C.); 2 Department of Architectural Engineering, Sejong University, Seoul 05006, Korea; mksung@sejong.ac.kr (M.S.); joseongmin01@naver.com (S.J.)

**Keywords:** transmission routes, evaporation, computational fluid dynamics, Middle East respiratory syndrome, cough droplets

## Abstract

Identifying infection transmission routes in hospitals may prevent the spread of respiratory viruses and mass infections. Most previous related research focused on the air movement of passive tracers, which typically represent breathing. In this study, particle evaporation and dispersions with various particle sizes were applied to evaluate particle movement because of breathing and coughing using computational fluid dynamics (CFD) simulations. Pyeongtaek St. Mary Hospital, where a Middle East respiratory syndrome (MERS) index patient infected several patients on the same floor, was used for a case study. We compared the dispersion characteristics of various particle sizes and validated results by comparing infection rates in different ward. Results indicated that droplets spread across the corridor and dispersed to wards that were more than 17 m apart from the index patient by natural ventilation. Droplets from exhaled breath under steady-state simulation showed a wider range of dispersion than cough droplets under transient simulation, but cough droplet dispersion was more consistent with the actual infection rate in each ward. Cough droplets sized under 75 µm evaporated to 26% of the initial size and started to disperse into the corridor within one minute; in nine minutes, droplets dispersed throughout every ward. This study may increase awareness on the dispersion characteristics of infectious particles.

## 1. Introduction

After the first case of COVID-19 was identified in December 2019, COVID-19 spread worldwide during the following months. As of 9 December 2021, there have been 267 million confirmed cases and 5.28 million deaths reported to the WHO globally [1]. There have been 12 cases of nosocomial clusters, wherein more than 100 cases of COVID-19 were confirmed at healthcare facilities in Korea. After the first case of COVID-19 in Washington, USA in January 2020, cluster infections in long-term care facilities, assisted-living facilities, and nursing homes fueled substantial community transmissions [2]. Due to the nature of respiratory infectious diseases, healthcare-associated infections in populated places with patients who are elderly, immunocompromised, or have underlying conditions have worse consequences than community infections. In addition to health vulnerabilities, these individuals are also exposed to infection because they live together in a closed space.

Respiratory viruses such as SARS-CoV, influenza A virus, MERS-CoV, and SARS-CoV-2 are known to be transmitted through droplets or direct and indirect contact with an infected person. Airborne diseases can be transmitted when pathogens present in the respiratory tract of an infected person are disseminated in salivary droplets during coughing, sneezing, talking, and breathing [3]. Droplets evaporate into pathogens, and the pathogens may remain airborne for some time, during which time they may be inhaled or ingested by a new host [3]. Direct and indirect contact transmission occurs when there is physical contact between an infected person and a susceptible person, or between a susceptible person and contaminated objects or surfaces [3].

Respiratory droplets are emitted during sneezing, coughing, speaking, and breathing. Sneezing and coughing are violent respiratory events that yield relatively high concentrations of large droplets [4]. Coughing is a common symptom of respiratory infections and is known to cause droplet transmission. Small droplets expelled during breathing can also be infectious [5]. The particles emitted during breathing are generally less than 1.0 µm in size [6,7,8]. Small droplets persist in the air for long periods and have a high probability of penetrating into the lower respiratory tract of a susceptible individual [6].

Previous studies have conducted simulations of breathing and coughing to analyze the transmission of respiratory viruses using CFD. Exhaled breath was simulated under steady-state conditions as breathing emits a certain amount of air volume over time. Li et al. [9] performed a numerical simulation to identify the transmission route of severe acute respiratory syndrome (SARS) in Hong Kong during the SARS epidemic in 2003 by assuming virus-containing bio-aerosols as a passive tracer which suggested the possibility of airborne transmission in hospital ward. Jo et al. [10] dispersed a passive tracer in a hospital where the first MERS-infected patient in Korea was admitted in 2015 by assuming the index patient’s breath would have the same dispersion as the tracer. The results indicated that the passive tracer from the index patient spread across the corridor and into other wards, which were 17.8 m apart from the index patient, by the outdoor airflow. Ou et al. [11] performed numerical simulations to investigate the possibility of airborne transmission in buses depending on the ventilation rate by assuming the exhalation of a COVID-19 patient as a tracer gas. They found that ventilation rates and exposure time affect the infection rates of passengers on the bus. Li et al. [12] also approximated the exhaled droplet nuclei as a passive scalar and analyzed an infection case that occurred in a restaurant in Guangzhou. The results showed that airborne transmission was possible in crowded spaces with poor ventilation.

Transient simulations have been conducted to simulate cough jets because the cough flow rate is time-dependent [13,14,15]. Mirzaie et al. [16] conducted a numerical simulation of cough droplet dispersion for an infected person in front of a classroom. They found that droplet concentration was lower with higher ventilation rate and the partitions installed in front of the seats. Zee et al. [17] performed CFD simulations to track cough particles expelled by passengers in an aircraft cabin and found that decreasing airflow caused an increase in particle dispersion throughout the cabin. These studies have suggested the possibility of infection through particle dispersion in the air.

Previous studies that investigated the transmission of infections in hospitals have focused on indoor airflow and passive tracer diffusion under steady-state conditions [9,10,18,19]. A passive tracer model can simulate the distribution of contaminated air; however, the effects of particle size, evaporation, and deposition are neglected [11,12]. Moreover, breathing and coughing are different respiratory activities. Hence, both breathing and coughing particle dispersion should be evaluated to analyze the spreading characteristics and clearly identify the cause of infection. However, a comparative analysis of breathing and coughing respiratory events has yet to be conducted, especially in large hospitals.

In this study, the dispersion characteristics of various particle sizes were investigated to evaluate the particle dispersion of droplets emitted during breathing and coughing. Exhaled breath droplets were simulated under steady-state and transient conditions to observe the difference between the two analyses, and cough droplets were simulated under transient conditions to assess the evaporation of droplets. In addition, the model was validated by comparing the actual incidence of infected patients and particle dispersion rate in each ward at the Pyeongtaek St. Mary’s Hospital, where the first MERS-infected patient in Korea was admitted in 2015.

## 2. Methods

### 2.1. Simulation Setup

**Figure 1:**
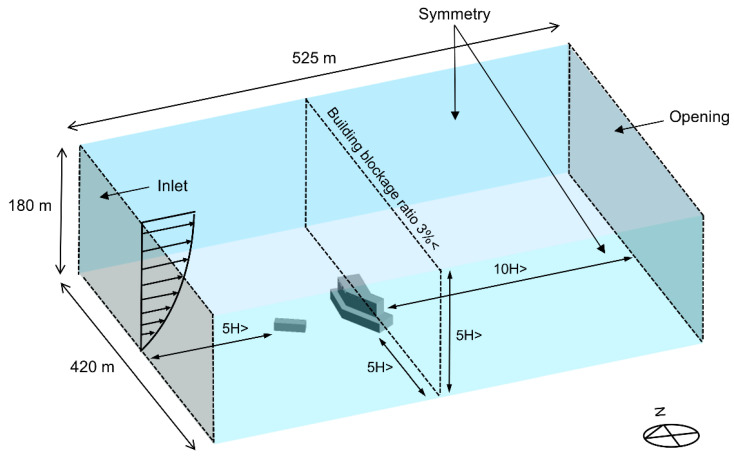
Domain for outdoor CFD simulation.

In order to conduct detailed evaluations for observing the characteristics of particle dispersion of breathing and coughing in realistic conditions, the boundary conditions of numerical simulations were based on an in-hospital MERS outbreak in Pyeongtaek, Korea, in May 2015. To assess the indoor airflow from the outside, the exterior of the hospital building, and surrounding obstruction, a 4-story building located 40 m to the west of the hospital was modeled as shown in Figure 1. There was sufficient space between the hospital building and analysis domain to allow the full development of airflow for obtaining reliable analysis results [20]. For boundary conditions, the inlet was set according to the direction of the wind, and the opening condition on the opposite side of the inlet was established with the atmospheric pressure at which the air exited, owing to the pressure built from the wind. Symmetry was assumed for the two lateral sides and the top side.

According to the weather data in Pyeongtaek between 15 and 17 May 2015, when the index patient was admitted, west and west-southwest winds at a temperature of 17.8 °C were prevalent, and the wind velocity at 10 m above ground was 2.6 m/s for the west wind and 2.62 m/s for the west-southwest wind [21]. And the average relative humidity was 67.2%. Thus, two separate inlet conditions were set for the west and west-southwest winds for validation of this simulation model. The vertical distribution of wind velocity was established according to an exponential law based on the wind velocity 10 m above the ground level. The roughness of the earth’s surface, the α value, was set to 0.22 (for regions where houses with a height of 3.5 m are concentrated or regions where middle-class buildings are scattered) according to the Korea Building Code [22].

**Figure 2:**
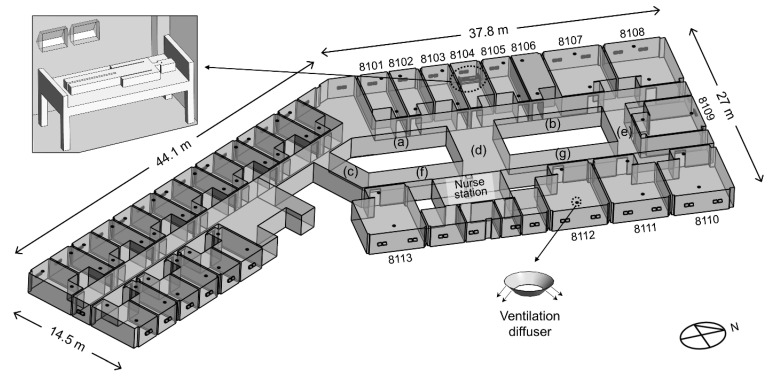
Domain for indoor model of the 8th floor. Corridors (a), (c), and (f) are located on the south side of ward 8104. Corridor (d) is the central corridor which is located between ward 8104 and the nurse station. Corridors (b), (e), and (g) are located on the north side of ward 8104.

The domain for the indoor model of the 8th floor is shown in Figure 2. The floor area of ward 8104 was 20 m^2^, and that of corridors (a)–(g) was 240 m^2^. The height of the 8th floor was 2.4 m. According to the epidemiological investigation at the time, all windows and doors on the 8th floor were set to be open. Air supply diffusers were installed close to the windows, and the exhaust air vents were located near the door of each ward on the 8th floor. However, ward 8104, where the index patient was hospitalized, did not have a ventilation system because the hospital was being remodeled. The ventilation rate for single-occupancy, double-occupancy, and seven-occupancy rooms is 200, 250, and 500 cubic meters per hour (CMH), respectively, representing approximately five air changes per hour (ACH). The bathroom was not modeled for each ward, but an outlet boundary of 100 CMH was placed on the undercut of the bathroom doors, thereby allowing airflow due to ventilation in the bathroom [10]. The index patient was modeled in ward 8104, lying near the window. The hydraulic diameter of the mouth opening was 10 mm [14]. The temperature of the index patient was assumed to be 34 °C.

The CFD analysis cases were selected based on the wind direction, mechanical ventilation system, particle expulsion type, and analysis method as shown in Table 1. In Case SA-1, a steady-state analysis of exhaled breath particles with no wind was conducted to implement a general situation. In Case W-1T, dispersion of exhaled breath droplets was simulated in a transient state. Cases of W-1, W-2, W-3, WSW-1, WSW-2, and WSW-3 considered exhaled breath droplets under steady-state conditions, as is commonly assumed for breathing simulations. Cases W-1C and WSW-1C considered cough particles expelled from the index patient in a transient state. In Cases SA-1, W-1T, W-1, W-1C, WSW-1, and WSW-1C, the mechanical ventilation system was not operated only in ward 8104, representing the actual situation when the index patient was hospitalized. In Cases W-2 and WSW-2, all ventilation systems were not in operation. In Cases W-3 and WSW-3, all ventilation systems, including that in ward 8104, were in operation.

### 2.2. Boundary Conditions for Breathing and Coughing Situations

Particle dispersion from the breathing and coughing of the index patient were investigated. The surrounding air was assumed to be an ideal mixture of dry air and water vapor. The air temperature of the exhaled air was assumed to be 35 °C [23], and the relative humidity was set at 90% [24]. Droplets consist of 1.8% non-volatile solid components and 98.2% water [13,15,25]. The water in the droplets gradually evaporates into solid droplet nuclei [13,26]. The droplet transport and trajectories were tracked using Lagrangian particle tracking. A multi-component Eulerian–Lagrangian model was used in this study to model the evaporation of droplets [13]. The airflow field was solved using Reynolds-averaged Navier–Stokes equations. Two-way coupling was employed, wherein the mass, momentum, and energy of the droplets were exchanged with the fluid phase [27]. Due to the low volume fraction of the droplets and low mass loading, droplet–droplet interactions were not considered [28]. Droplet deposition occurred once the droplet contacted the surface.

The equation of motion for a particle in a continuous fluid is defined as:

(1) $$m_{p}\frac{dU_{p}}{d_{t}}=F_{D}+F_{B}+F_{R}+F_{VM}$$

where $m_{p}$ is the droplet particle mass, $U_{p}\;$ is the droplet velocity, and $F_{D}\;$ is the drag force acting on the particle from the surrounding vapor. $F_{B}$ is the buoyancy force due to the gravity, $F_{R}$ is the forces due to domain rotation, and $F_{VM}$ is the virtual mass force.

The evaporation of water is controlled by the equilibrium vapor pressure relative to the ambient pressure at the droplet surface [13,15]. The equilibrium vapor pressure at the droplet surfaces can be estimated by Antoine Equation:

(2) $$P_{vap}=P_{scale}\exp\left(A-\frac{B}{T+C}\right)$$

where $P_{scale}$ is the pressure scale used to scale the units for the vapor pressure. When $P_{scale}=$1.0 bar, the Antoine reference state constant *A* of 12,439, the Antoine reference state constant *B* of 4233.7, and the Antoine temperature coefficient *C* of −31.737 [29].

In common indoor air, the mass transfer rate is given by [13,15]:

(3) $$\frac{dm_{d}}{dt}=-\frac{dS_{v}}{dt}=-\pi d_{d}\rho D\mathrm{Sh}\frac{W_{v}}{W_{m}}\ln\left(\frac{P-P_{v,s}}{P-P_{v,m}}\right)$$

where $d_{d}$ is the droplet diameter, ρD  is the dynamic diffusivity of water vapor in the continuum, and Sh is the Sherwood number [30]. $W_{v}$ and $W_{m}$ are the molecular weights of the vapor and the mixed air. *P* is the ambient pressure, $P_{v,s}$ and $P_{v,m}$ are the partial pressures of water vapor at the droplet surface and in the air mixture, respectively.

**Figure 3:**
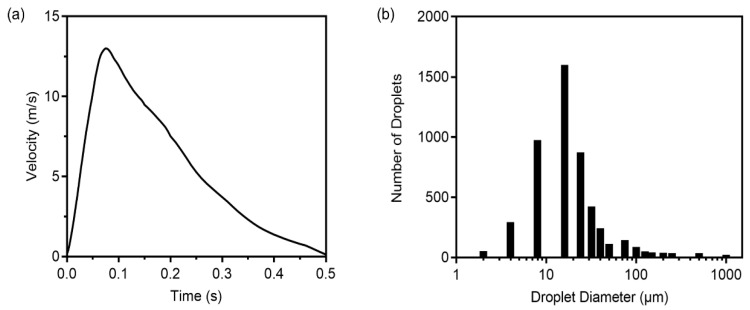
(**a**) Airflow velocity of droplets from a single cough; (**b**) Cough droplet size distribution.

A single cough behavior was considered, with a duration of 0.5 s and a peak air velocity of 13 m/s at 0.08 s, as shown in Figure 3a [4,31,32]. The total number of expelled cough droplets was 4897, and droplets ranged in diameter from 2 to 1000 µm, as shown in Figure 3b [33]. A transient simulation for cough was conducted for 900 s.

Exhalation flow in a transient state is expressed by the following equations [34]:

(4) $$V_{sin}=4.5\sin(1.79t)$$

(5) $$V_{e}=\{\begin{array}{c} 0\;if\;v_{sin}<0\\ v_{sin}\left(t\right)\;if\;v_{sin}>0 \end{array}\;$$

where the maximum velocity of exhalation is 4.5 m/s and inhalation was ignored. The number of expelled droplets in exhaled air was 2 particles/cm^3^ [35]. A transient simulation for breathing was conducted for 40 min. The average rate of the index patient’s breathing under steady-state conditions was set to 0.27 CMH [36], with the same number of cough droplets, each of a size of 0.4 µm.

The model equations were solved using the commercial CFD code CFX, with the shear stress transport (SST) model for the air turbulence [37]. Mesh independence was achieved using 12,046,731 mesh elements. The models were solved under the root mean square (RMS) residual convergence criteria of 1 × 10^−4^. The initial conditions for transient calculations were obtained from steady simulations.

## 3. Results and Discussions

### 3.1. Analysis of Breathing Particle Dispersion

**Figure 4:**
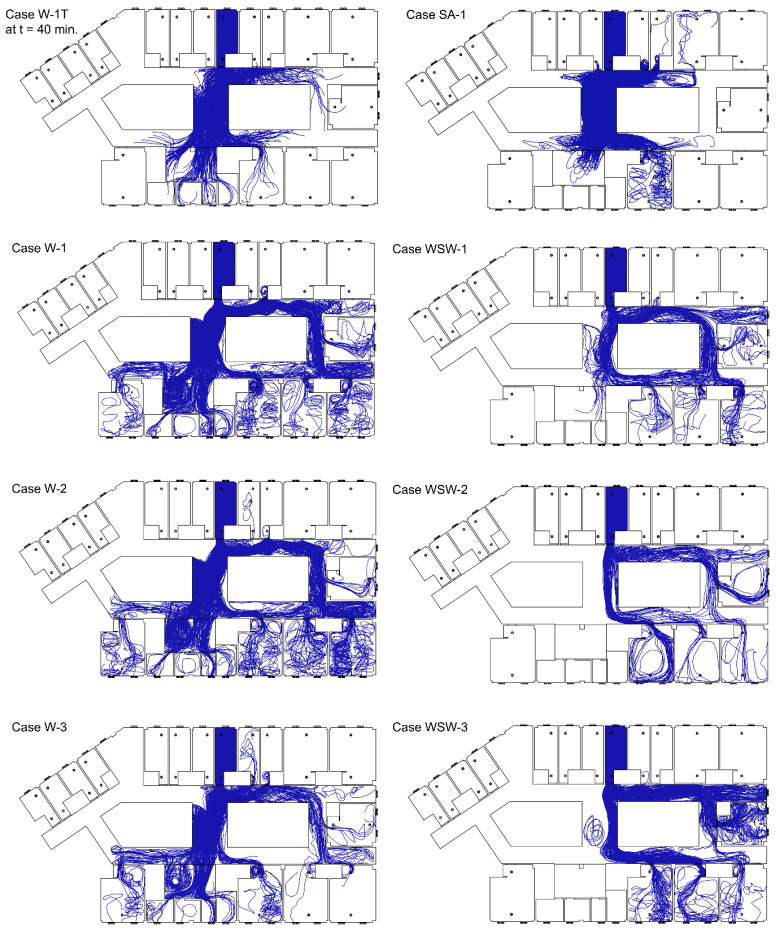
Trajectories of particles emitted from breathing.

Droplets expelled from breathing were investigated under transient and steady-state conditions. Figure 4 shows the particle trajectories of exhaled breaths from the index patient. Expelled droplets evaporated to 0.1 µm, which is approximately 26% of their initial size. Case W-1T showed a short range of particle dispersion than other cases that simulated west wind in steady-state conditions because a small number of droplets were expelled over time. It appears that steady-state analysis demonstrated more extreme results of particle dispersion than transient analysis. In Case SA-1, droplets dispersed to the corridor and moved to wards 8103, 8105, 8106, and 8107, where the location is near ward 8104, by the induced airflow from the exhaust air vents near the door. Droplets also spread to the corridor (d) and dispersed to the nurse station and ward 8112. The particle dispersed more with mechanical ventilation in static air.

With the pressure difference from the wind, droplets moved longer distances. In the case of the west wind, droplets dispersed mainly to corridor (d) and moved to the nurse station, and droplets that dispersed to corridor (g) moved to wards 8110, 8111, and 8112. Some droplets dispersed in corridor (f) and spread to ward 8113. In addition, droplets dispersed to corridor (b) spread to wards 8105 and 8106. In the case of ward 8106, where there were no infected patients, droplets moved back to the corridor after moving through the door. The droplets moved to corridor (e) through corridor (b) and spread to wards 8109 and 8110. In the case of west-southwest wind, droplets moved to corridor (d) and corridor (g), and then dispersed in wards 8110, 8111, and 8112. Some droplets moved to corridor (b) and spread to wards 8105 and 8106, while others moved to corridor (e) and spread to wards 8109 and 8110.

**Figure 5:**
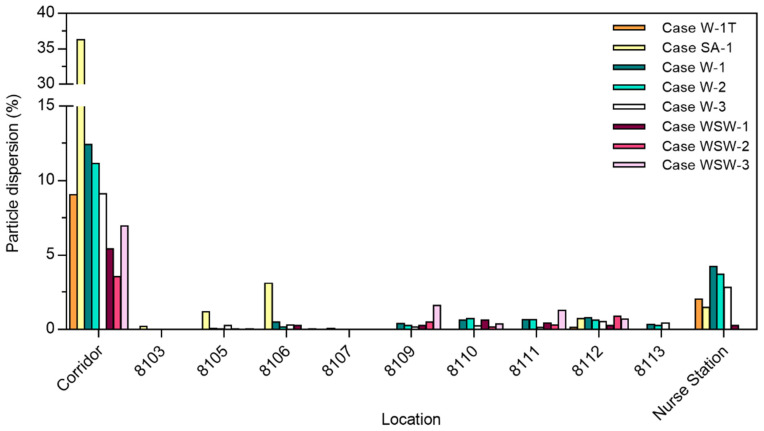
Particle dispersion to different wards and in the corridor.

The particle dispersion rate in each ward is shown in Figure 5. In Case SA-1, more droplets dispersed to the corridor than in other cases with the wind. This is because there was no strong indoor airflow induced by the external wind and few droplets deposited in ward 8104. The west-southwest wind led to a greater deposition of droplets than west wind, leading to greater dispersion to the corridor in the case of west wind. When mechanical ventilation was in operation, the airflow induced from the window was affected by the air supply from the air vent near the window. Outdoor airflow by west wind induced indoor airflow toward the west from the window in ward 8104. In Case W-3, the airflow toward the west was disturbed by the air supply, so particles could not disperse directly to the corridor, with many particles being deposited in ward 8104. In Case WSW-3, few droplets deposited on the northern wall because of poor airflow toward the north, resulting in the dispersal of more droplets into the corridor. Therefore, the droplet dispersion pattern in the case of open windows and doors appears to be more affected by the direction of wind from the outside than by the ventilation system.

In Cases W-1T, SA-1, W-1, W-2, W-3, WSW-1, WSW-2, and WSW-3, the total droplet dispersion rate in each ward was 2.2%, 6.7%, 7.6%, 6.4%, 4.8%, 2.1%, 1.8%, and 4.0%, respectively. Compared with Cases W-2 and WSW-2, Cases W-1 and WSW-1 had a higher particle dispersion rate because the exhaust air vents located near the door induced airflow into each ward.

### 3.2. Analysis of Cough Particle Dispersion

**Figure 6:**
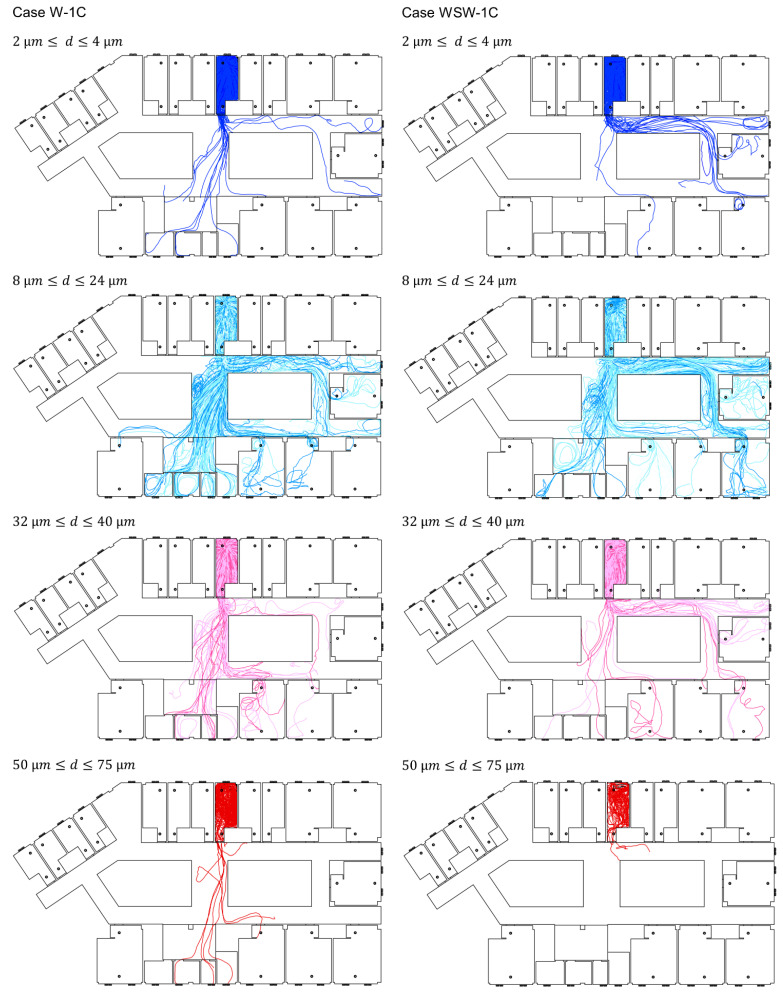
Trajectories of cough particles.

**Figure 7:**
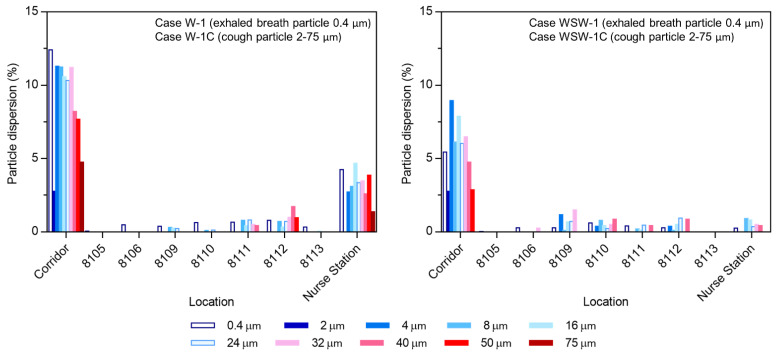
Particle dispersion rate with different wind directions.

Cough particle dispersion was investigated using transient analysis. Figure 6 shows the particle trajectories according to particle size, and Figure 7 shows the dispersion rate of exhaled breath particles (0.4 µm) and cough particles (2–75 µm) when the 4897 droplets expelled from the index patient were considered to be 100% of existing droplets. Although the size of the expelled droplets ranged from 2 to 1000 µm, only droplets smaller than 75 µm were analyzed, as droplets between 100 and 1000 µm did not disperse into the corridor. Most droplets dispersed in the corridor had sizes under 50 µm. These droplets evaporated to 13 µm within 2.3 s, and the smallest 2 µm droplets rapidly evaporated to 0.5 µm within 0.002 s.

In Case W-1C, a similar movement pattern was observed for droplet sizes of 8–24 µm, and most of the cough droplets spread to wards 8109, 8111, 8112, and the nurse station. Droplets with a size of 100 µm spread throughout ward 8104 but did not disperse to the corridor. Droplets larger than 100 µm fell near the index patient immediately after coughing. Of all expelled particles, 9.8% dispersed in the corridor, and 35% of the particles in the corridor infiltrated the nurse station.

Compared with Case W-1C, Case WSW-1C had a strong airflow movement to corridor (b) from corridor (a). Droplets with a size of 2 µm were deposited in the corridor, and 4 µm droplets spread to wards 8109, 8110, and 8112. Droplets with sizes between 8 and 24 µm spread to wards 8109–8112, and the nurse station. Droplets with a size of 50 µm dispersed in the corridor, but deposited near ward 8104. Droplets with a size of 75 µm spread across ward 8104 but did not disperse into the corridor. Of all expelled particles, 6.2% dispersed in the corridor, whereof 9.3% spread to the nurse station and 9.0% spread to ward 8109. Droplets larger than 100 µm experienced gravitational sedimentation and deposited near the index patient.

Cough droplets started to spread to the corridor in 73 s and 46 s in Cases W-1C and WSW-1C, respectively. After 100 s of droplet emission, 3.3% and 1.6% of droplets dispersed to the corridor in Cases W-1C and WSW-1C, respectively. In Case W-1C, droplets first dispersed to the nurse station in 170 s, and they dispersed to ward 8111 and 8112 within 250 s. The droplets then dispersed to ward 8113 in 480 s, and to ward 8110 in 525 s. In Case WSW-1C, droplets first dispersed to ward 8112 in 200 s. Droplets also dispersed to ward 8111 and the nurse station within 250 s. The droplets then dispersed to ward 8106 and 8109 within 300 s, and to 8110 in 340 s.

Small droplets (2–4 µm) dispersed less to the corridor and other wards, because of the small number of droplets of this size fraction. Droplets that mainly dispersed throughout the ward were droplets sized between 8 and 40 µm, and droplet sizes of 8–24 µm were transported through the air over long distances. Droplets sized between 50 and 75 µm could disperse to other wards after evaporation, provided there were no obstacles, such as bends, in the particle trajectories, while droplets larger than 100 µm fell near the index patient immediately after coughing.

Droplets expelled from the index patient started to disperse into the corridor after 1 min on average. In both cases, ward 8110 was the last ward where the droplets reached, and the time required differed by approximately 3 min depending on the wind direction. In less than 9 min, droplets dispersed into each ward of the 8th floor. Although it is important to reduce the concentration of infectious aerosols through natural ventilation, the central corridor structure of Pyeongtaek St. Mary’s Hospital and high natural ventilation rate caused the dispersion of infectious aerosols throughout the wards.

### 3.3. Comparison of Breath and Cough Particle Dispersion

The dispersion of exhaled breath particles under steady-state conditions and that of cough particles under transient conditions were investigated, and the results of the two droplet dispersions were compared. As shown in Figure 7, exhaled breath droplets showed a wider range of particle dispersion than cough droplets. In addition to ward 8109, 8110, 8111, 8112, and the nurse station, droplets also dispersed to wards 8105, 8106, and 8113 in Cases W-1 and to ward 8105 in Case WSW-1. The reason why breathing led to a wider range of particle dispersion than coughing is because the number of expelled particles was equal, while the particle size from breathing was 0.4 µm, which is less affected by gravitational sedimentation than cough particles (2–1000 µm). As all particles followed the established flow field, they showed similar results to the airflow dispersion.

**Figure 8:**
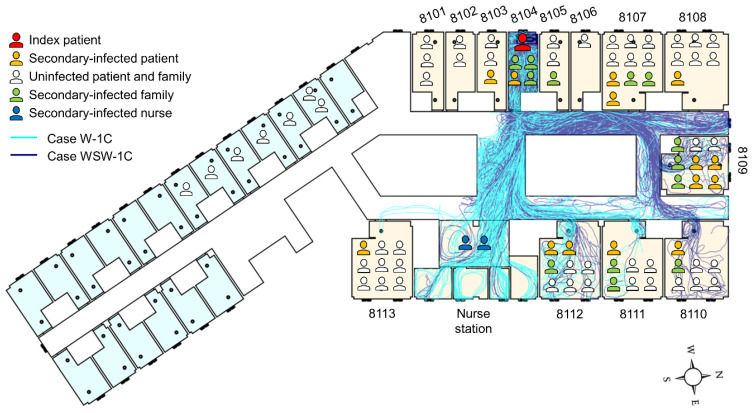
Schematic of MERS patients on the 8th floor of Pyeongtaek St. Mary’s Hospital with the trajectories of cough particles. Maternity ward and general ward are colored light blue and light brown, respectively.

Pyeongtaek St. Mary’s Hospital, which is the base model for this study, was where the first MERS-infected patient in Korea was hospitalized, in ward 8104 for three days in May 2015. As shown in Figure 8, no infected patients were reported in the maternity ward to the south. Some patients and family members in wards 8103, 8105, 8107, and 8108, which were adjacent to ward 8104, were infected. In addition, infection occurred in wards 8109–8113, and at the nurse station, which were more than 15 m away from ward 8104.

From the epidemiological investigation, 37 people were found to be infected at Pyeongtaek St. Mary’s Hospital. Of these, 16 patients (43.2%) were confirmed to have come into close contact with the index patient, and except for 9 patients (24.3%) whose movement path was close to the index patient, the transmission route of the remaining 9 patients was unclear. In addition, 7 out of 9 patients were patients and family members who were admitted to wards 8110–8113, and the infection rates were 29%, 43%, 38%, and 11%, respectively, which are high, considering the short period of time.

In both breathing and coughing cases with wind, particle dispersion did not occur in wards 8101, 8102, 8103, 8107, or 8108. Particles dispersed to ward 8109, where the most infected cases occurred, and to wards 8110–8113, where secondary patients were infected without close contact with the index patient. In particular, the nurse station and ward 8109 had a high infection rate and droplet concentration in Case W-1C and WSW-1C, respectively. This indicates the possibility of high particle concentration leading to a high infection rate.

**Figure 9:**
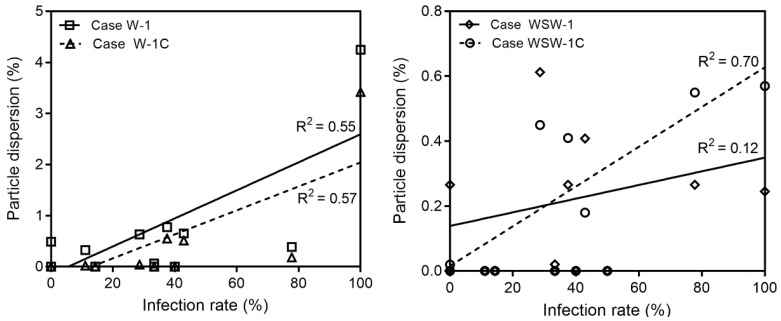
Validation for the accuracy of breathing and coughing simulation.

Combining the epidemiological investigation and particle dispersion analysis results, we found that transmission in wards 8103, 8107, and 8108 appears to have patients through close contact. Figure 9 demonstrates the infection rate of wards 8101–8113 and nurse station from the particle dispersion, which occurred from breathing and coughing in ward 8104. The infection rate is expressed as the percentage of infected people among people who were exposed to the risk of infection in each ward. Wards 8101, 8102, 8103, 8107, and 8108, where particle dispersion did not occur, appeared to have an infection rate of 0% on the *Y*-axis of the graph. The regression line and its R-squared values are expressed as the values of wards 8101–8113 and nurse stations, excluding wards 8103, 8104, 8107, and 8108. Compared to the R-squared values including wards 8103, 8107, and 8108, the R-squared values without these three wards increased in all cases, from 0.51 to 0.55 (Case W-1), 0.54 to 0.57 (Case W-1C), 0.05 to 0.12 (Case WSW-1), and 0.5 to 0.7 (Case WSW-1C). Therefore, it appears that the airborne transmission of infectious aerosols in wards 8109, 8110, 8111, and 8112, as well as at the nurse station, acted more strongly than direct or indirect contact transmission. In addition, when comparing the R-squared values of breathing and coughing particles in either wind direction, the value for coughing was higher. Therefore, when predicting the incidence of infections using CFD simulations, using particles emitted from coughing may be more reliable than using particles released from breathing.

The immune systems of patients, caregivers, and healthcare workers who were in Pyeongtaek St. Mary’s Hospital during the MERS outbreak were all different, and the possibility of fomite transmission could not be excluded. The particle dispersion rate at each ward determined in this study might be low, and there is a low correlation between the particle dispersion rate and incidence of infected patients. However, it is clear that the possibility of airborne transmission by indoor airflow existed.

This study confirmed the possibility of airborne transmission in hospitals, indicating that droplets can be dispersed by natural ventilation. However, uncertainties may occur from the positions and effects of occupants, hospital equipment, and furniture in wards, and constant wind direction and velocity. Future studies should consider the effects of the thermal plume of the human body and particle resuspension by human-induced wake. Various situations, such as changes in wind direction and speed during the simulation, could also be investigated. In addition, mechanical ventilation strategies in hospitals to minimize particle dispersion need to be investigated.

## 4. Conclusions

In this study, exhaled breath and cough particles were investigated and compared in terms of the particle dispersion in Pyeongtaek St. Mary’s Hospital, where the first MERS index patient was admitted in Korea in 2015. With the windows open, there was an influx of outside air into the room, and infectious particles from the index patient spread to adjacent wards and towards across the corridor.

The exhaled breath particles in steady-state conditions exhibited a wider range of dispersion than cough droplets. To identify a wide range of transmission routes, it would thus be more advantageous to analyze exhaled breath particles under steady-state conditions. However, when comparing the actual incidence of infection with the dispersion rate at each ward, the simulated cough particle dispersion showed higher reliability than exhaled breath particles. Therefore, it is necessary to investigate both exhaled breath and cough particles when investigating transmission routes.

Droplets evaporate to approximately 26% of their initial size during dispersion. Droplets dispersed throughout the wards were small (≤4 µm) and medium (8–75 µm) in size. Small droplets did not have much of an effect on particle dispersion because of the small number of droplets. Droplet sizes of 8–24 µm are transported through the air over long distances. Large droplets (>100 µm) fell near the index patient immediately after being expelled.

Droplets started to disperse into the corridor within 1 min, and in 9 min, droplets dispersed throughout the ward. Therefore, it is necessary to establish preventive measures at the initial phase, when the hospitalized patient is confirmed to be infected, because viruses can rapidly spread throughout a hospital floor.

## Figures and Tables

**Table 1:** CFD simulation cases.

	Wind Direction	Mechanical Ventilation System	Respiration Event	Analysis Method
Case SA-1	Static air	Operated except in ward 8104	Breathing	Steady-state
Case W-1T	West			Transient
Case W-1C			Coughing	
Case W-1			Breathing	Steady-state
Case W-2		Not operated in all wards		
Case W-3		Operated in all wards		
Case WSW-1C	West-southwest	Operated except in ward 8104	Coughing	Transient
Case WSW-1			Breathing	Steady-state
Case WSW-2		Not operated in all wards		
Case WSW-3		Operated in all wards		
